# Thoracic Compressive Myelopathy in a Patient With Takayasu Arteritis

**DOI:** 10.7759/cureus.33496

**Published:** 2023-01-08

**Authors:** Nandyal Chandrasekar, Manikandan Thandapani, Ramachandran Govindasamy, Satish Rudrappa, Balaji Vaithialingam

**Affiliations:** 1 Department of Neurosurgery, Sakra World Hospital, Bengaluru, IND; 2 Department of Spine Surgery, Sakra World Hospital, Bengaluru, IND; 3 Department of Anaesthesiology, Sakra World Hospital, Bengaluru, IND

**Keywords:** spastic paraplegia, immunotherapy, decompressive laminectomy, takayasu arteritis, compressive myelopathy

## Abstract

Takayasu arteritis (TA), also known as occlusive thromboaortopathy, is a type of chronic inflammatory arteritis that primarily affects large vessels. Compressive thoracic myelopathy is a rare and distinct manifestation of TA. We present the case of a 60-year-old woman who developed gradually progressive spastic paraplegia over one year. Magnetic resonance imaging revealed a well-defined extra-dual, intensely enhancing ventrodorsal lesion with severe spinal cord impingement. The aortogram revealed dilatation of the aortic arch (with narrowing of arch vessels) and descending aorta, as well as a right paravertebral soft tissue mass at the D4 level. Given the likelihood of TA, the patient underwent decompressive laminectomy and spinal fusion due to severe spinal cord compression. The biopsy of the dural-based lesion revealed an inflammatory granuloma, and the patient was treated postoperatively with oral prednisolone and mycophenolate mofetil. After six months of immunotherapy, there was excellent neurological recovery and near-total resolution of the lesion.

## Introduction

Takayasu arteritis (TA) is a systemic inflammatory disease marked by granulomatous inflammation of large vessels. Large vessel vasculitis most commonly affects the aorta and its major branches, resulting in arterial occlusion and thrombosis and thus ischemic symptoms. It is reported to affect 2.6 million people per year in North America [1]. We present a rare case of TA with thoracic compressive myelopathy that was successfully treated surgically and with immunosuppressive therapy.

## Case presentation

**Figure 1:**
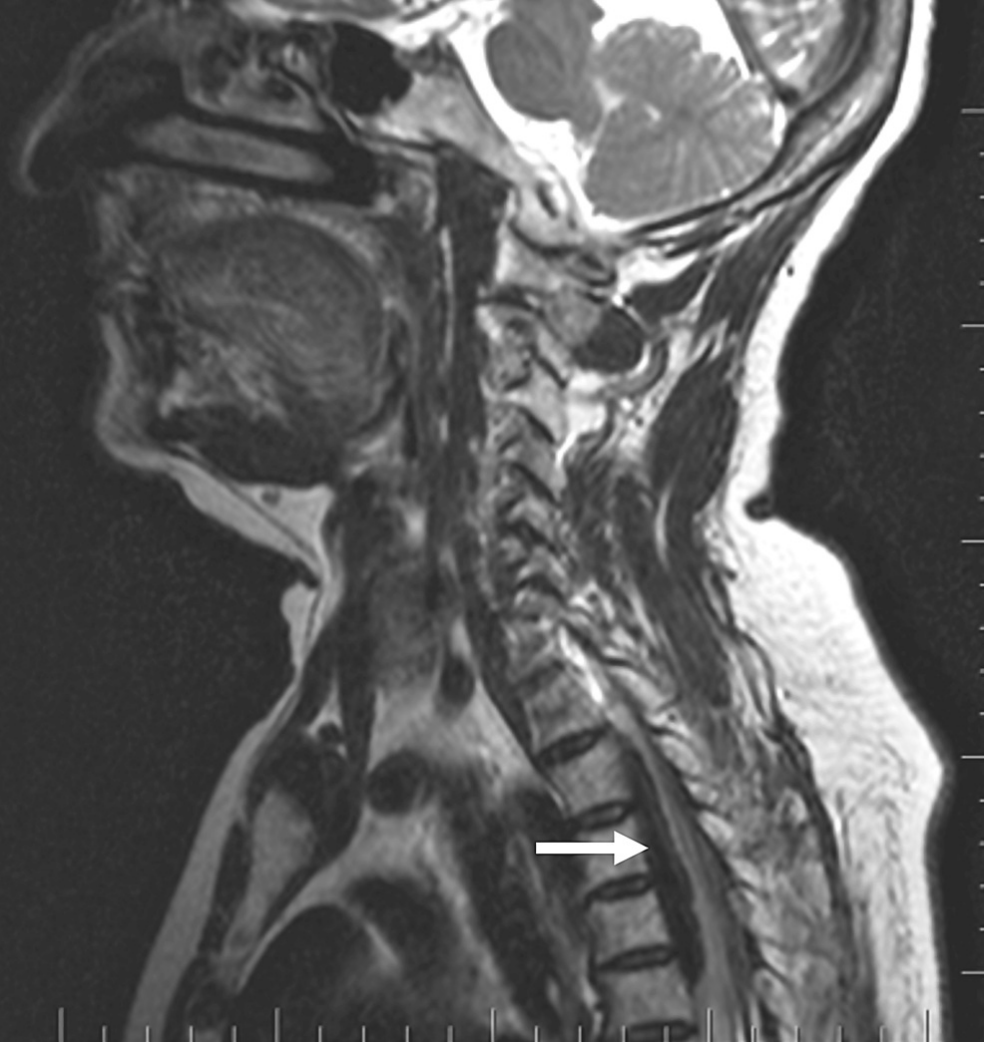
Preoperative MRI (T2 sagittal) showing a well-defined extradural and ventrodorsal lesion causing severe impingement of the spinal cord (arrow).

**Figure 2:**
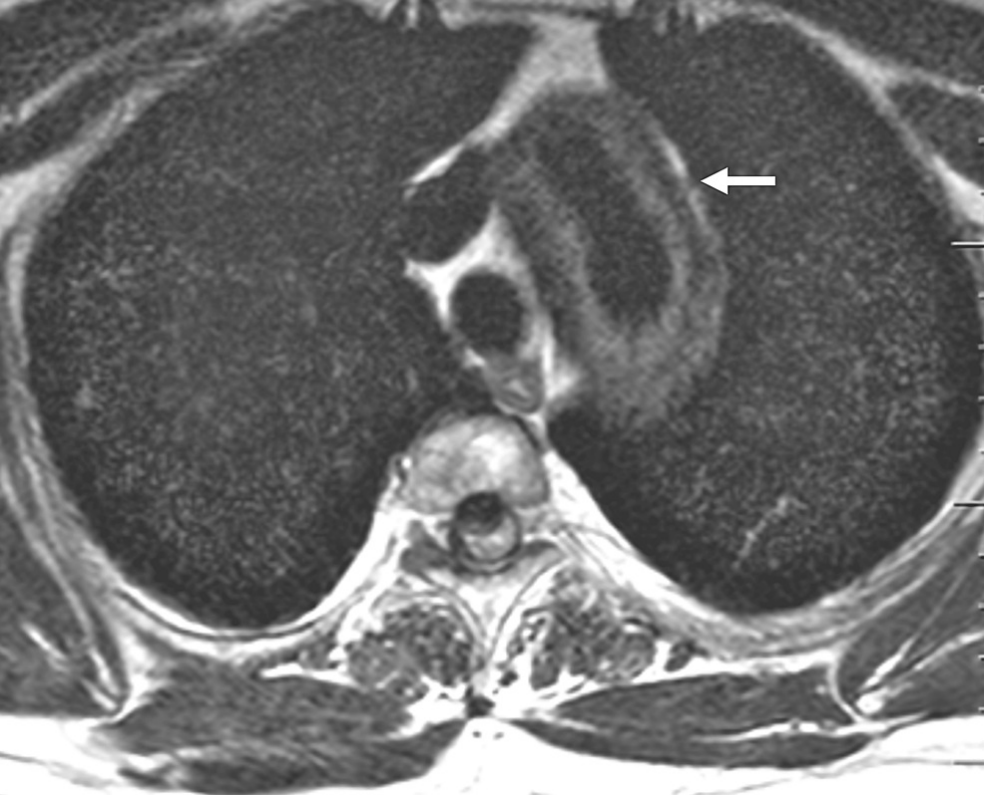
Preoperative MRI (T2 axial) showing a dilated thoracic aorta with a periaortic soft tissue mass (arrow).

A 60-year-old diabetic woman presented to the emergency department with lower-limb weakness and loss of sensation that had developed gradually over a year along with upper back pain. A history of trauma, tuberculosis, fever, weight loss, and bladder and bowel incontinence were all ruled out. There was no prior history of hospitalisation or medical treatment. The patient was fully conscious with a Glasgow coma score (GCS) of 15. Mild tenderness at the D4-D5 vertebral level with spastic paraplegia (power 0/5) with no upper extremity weakness and an exaggerated deep tendon reflex was noted on clinical examination. A further neurological examination revealed that pinprick and fine touch sensations were reduced below the level of D5, but posterior column sensations were intact. The magnetic resonance imaging (MRI) revealed a well-defined extradural, intensely enhancing right-sided ventrodorsal semicircular lesion causing severe spinal cord impingement (Figure 1) as well as a periaortic soft tissue mass (Figure 2).

**Figure 3:**
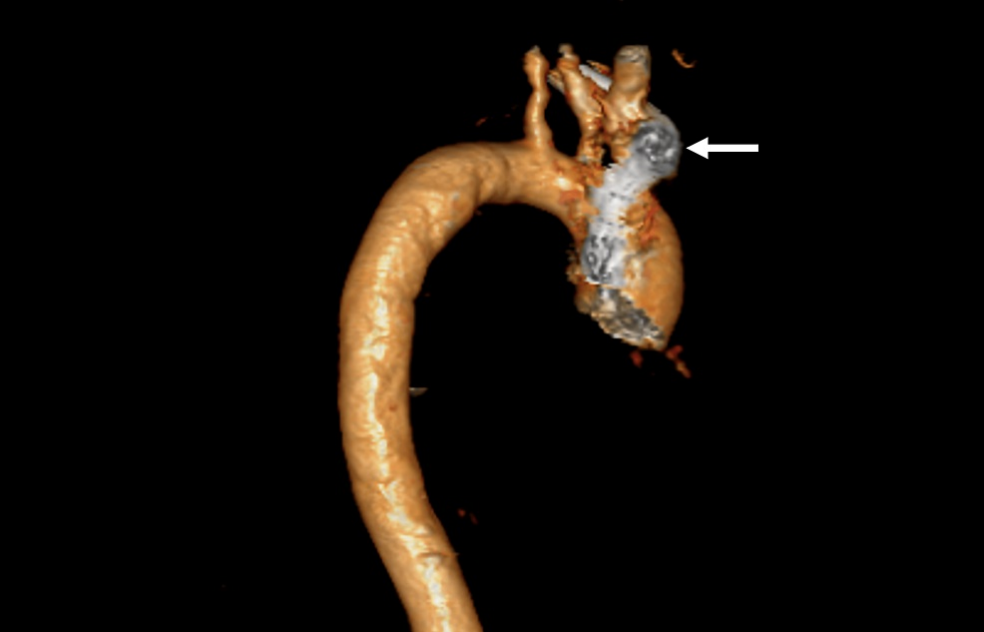
Preoperative CT-aortogram (3D) showing a dilated aorta with a periaortic mass lesion (arrow).

The lesion was found to have a ventral extension from D1-D5 and a dorsal extension from D2-D6. The CT aortogram revealed diffuse thickening and enhancement of the ascending aorta, aortic arch, and descending aorta, with periaortic mass measuring up to 13 mm (Figure 3).

**Figure 4:**
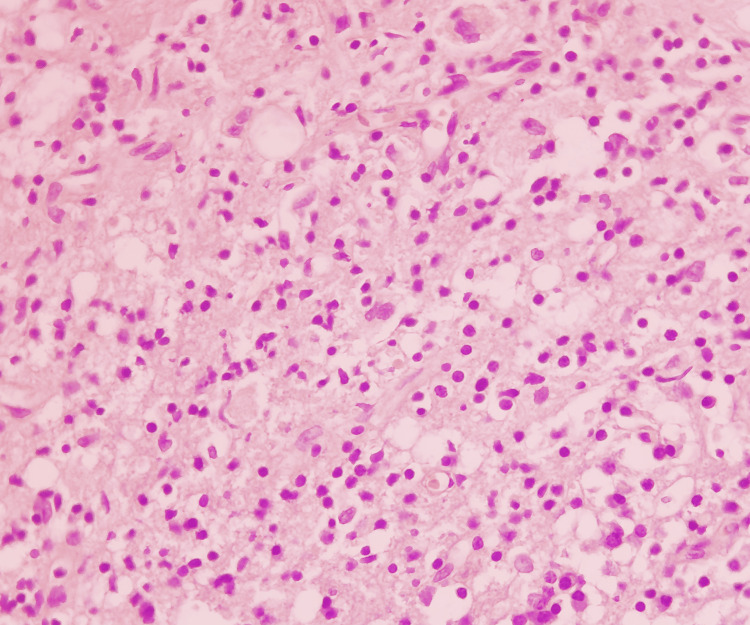
Hematoxylin-eosin stain (x400) of the tissue specimen from the dural mass lesion showing a dense chronic inflammatory infiltrate composed of foamy histocytes, lymphocytes, and plasma cells.

The proximal parts of the aortic arch vessels, bilateral renal arteries, and common iliac vessels were also thickened with luminal narrowing. A repeat clinical examination revealed a lower radial pulse intensity on the right upper limb compared to the left, with a systolic pressure difference of 20 mmHg. There was no tenderness or thickening of the bilateral superficial temporal arteries on palpation. Preoperative investigations revealed elevated erythrocyte sedimentation rate (ESR) (74 mm/hr) and C-reactive protein (2.9 mg/dl) with elevated neutrophils (88% on the differential blood count). Anti-dsDNA (18 IU/ml), anti-cardiolipin immunoglobulin G (IgG) (<2 U/ml), and rheumatoid factor (8.6 IU/ml) levels were normal. The rapid plasma reagin test was non-reactive, and anti-nuclear antibody results were negative. The viral markers (HIV, hepatitis B surface antigen (HBsAg), and hepatitis C virus (HCV)) were also non-reactive. Based on large vessel vasculitis features on imaging and European League Against Rheumatism (EULAR) diagnostic criteria for TA, a probable diagnosis of TA leading to compressive thoracic myelopathy was made, and the patient was immediately scheduled for surgical decompression due to severe spinal cord compression. A biopsy of the lesion was performed after transpedicular decompression at the D4-D5 level, followed by decompressive laminectomy (D1-D6) and posterior fusion with transpedicular screws. Histopathological examination of the tissue revealed a dense chronic inflammatory infiltrate composed of foamy histocytes, lymphocytes, and plasma cells (Figure 4).

**Figure 5:**
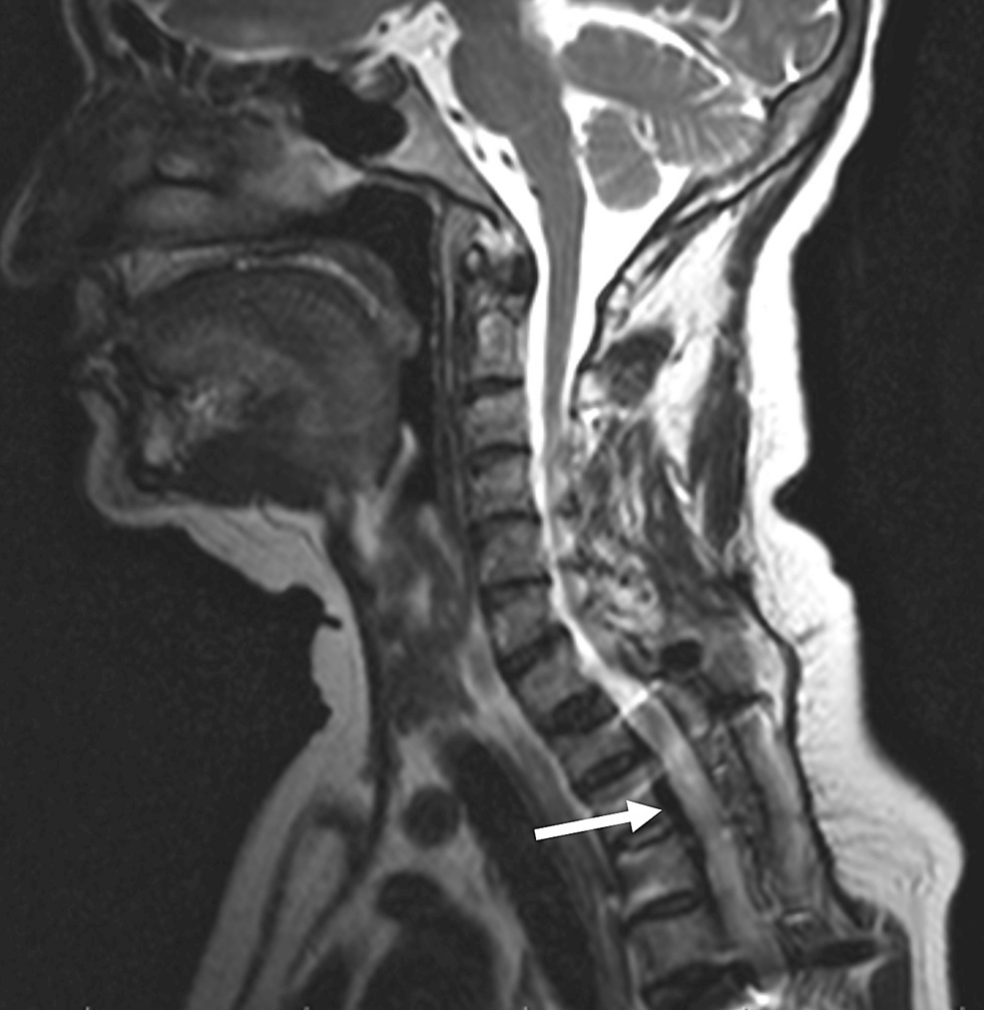
Postoperative MRI (T2 sagittal) showing near-complete resolution of the extradural mass lesion (arrow).

The acid-fast bacilli stain was negative, and there was no evidence of necrosis. The probability of IgG4-related disease was also ruled out, as the immunohistochemistry staining of the biopsy specimen revealed <10% IgG4 plasma cells with absent storiform fibrosis and phlebitis. Because there was no improvement in the patient's neurological status in the immediate postoperative period, the patient was advised to undergo rehabilitation before being discharged from the hospital. To treat large vessel vasculitis caused by TA, the rheumatologist prescribed oral prednisolone 20 mg/day and mycophenolate mofetil 1500 mg/day. The immunotherapy was gradually tapered over six months, and a six-month follow-up MRI revealed a near-complete reduction in the extradural mass (Figure 5) as well as full neurological recovery (Power 5/5).

## Discussion

As a result of steno-occlusive lesions and thromboembolism, TA can cause a variety of cerebral manifestations [2]. Dizziness, stroke, and transient ischemic attacks are common clinical manifestations. The involvement of the spinal cord due to thoracic inflammatory epiduritis can be an unusual manifestation of TA. The paraaortic inflammatory soft tissue mass that extends into the spinal epidural space can mimic a dural-based lesion and cause thoracic compressive myelopathy. We ruled out the diagnosis of giant cell arteritis because there was no evidence of temporal arteritis and jaw tenderness on clinical examination. The diagnosis of transverse myelitis (TM) was also ruled out based on the fact that TM has an acute presentation with bladder incontinence. The presence of a dural mass lesion was also against the diagnosis of TM. A dilated aorta should alert the physician to the presence of aortitis, and further clinical evaluation should be performed to determine the aetiology. We made the diagnosis of TA based on the classic imaging findings and EULAR criteria [3]. Steroids, along with other conventional immunosuppressive agents such as methotrexate, azathioprine, cyclophosphamide, and mycophenolate mofetil, are the mainstay of treatment in TA [4]. Acute spinal cord compression may necessitate an emergent surgical decompression procedure, such as a decompressive laminectomy. There is limited literature evidence on the management of TA with thoracic myelopathy. In a patient with TA who presented with acute spinal cord compression, Kim et al. performed an emergent decompressive laminectomy as well as excision of the dural mass lesion [5]. In the postoperative period, oral steroids, cyclophosphamide, and azathioprine were started, and good neurological recovery was observed over time. Similarly, Murugan et al. performed an emergent decompressive laminectomy with epidural mass excision in a patient with TA-induced spinal pachymeningitis [6]. With high-dose steroids, the patient showed excellent neurological recovery in the postoperative period. As a result, surgical decompression may be a viable option in acute spinal cord compromise in TA, but its role in chronic thoracic myelopathy is debatable. The patient presented with a chronic course of paraplegia that progressed gradually over a year, and a decompressive laminectomy with a lesion biopsy was performed to confirm the diagnosis. In our case, the combination of steroids and mycophenolate mofetil resulted in total neurological recovery and near-complete resolution of the mass lesion on follow-up imaging at six months.

## Conclusions

Compressive myelopathy secondary to the paraaortic inflammatory mass lesion can be an unusual manifestation of TA that can mislead the surgeon toward an intrinsic spinal pathology. Emergency decompressive laminectomy with postoperative immunosuppressive therapy can provide optimal neurological recovery.
